# Autophagy Regulation Influences β-Amyloid Toxicity in Transgenic *Caenorhabditis elegans*

**DOI:** 10.3389/fnagi.2022.885145

**Published:** 2022-05-12

**Authors:** Hongru Lin, Yehui Gao, Chen Zhang, Botian Ma, Mengchen Wu, Xianghuan Cui, Hongbing Wang

**Affiliations:** Putuo People’s Hospital, School of Life Sciences and Technology, Tongji University, Shanghai, China

**Keywords:** *Caenorhabditis elegans*, autophagy, RNA-sequencing, quantitative proteomics, Alzheimer’s disease

## Abstract

Alzheimer’s disease (AD) is a progressive, neurodegenerative disease characterized by the accumulation of amyloid-beta (Aβ) proteins in the form of plaques that cause a proteostasis imbalance in the brain. Several studies have identified autophagy deficits in both AD patients and AD animal models. Here, we used transgenic *Caenorhabditis elegans* to study the relationship between autophagy flux and Aβ. We labeled autophagosomes with an advanced fluorescence reporter system, and used this to observe that human Aβ expression caused autophagosome accumulation in *C. elegans* muscle. The autophagy-related drugs chloroquine and 3-MA were employed to investigate the relationship between changes in autophagic flux and the toxicity of Aβ expression. We found that reducing autophagosome accumulation delayed Aβ-induced paralysis in the CL4176 strain of *C. elegans*, and alleviated Aβ-induced toxicity, thus having a neuroprotective effect. Finally, we used RNA-sequencing and proteomics to identify genes whose expression was affected by Aβ aggregation in *C. elegans*. We identified a series of enriched autophagy-related signal pathways, suggesting that autophagosome accumulation impairs Aβ protein homeostasis in nematodes. Thus, maintaining normal autophagy levels appears to be important in repairing the protein homeostasis imbalance caused by Aβ expression.

## Introduction

Alzheimer’s disease (AD) is a devastating neurodegenerative disorder with no known cure (Savelieff et al., 2019). Multiple factors, such as amyloid-beta (Aβ) peptide aggregation (Selkoe and Hardy, 2016), tau-protein hyperphosphorylation (Mangialasche et al., 2010), inflammatory processes (Martin et al., 2017), and oxidative stress (Huang and Mucke, 2012), are considered to be causative of AD. Additionally, growing evidence suggests that autophagy deficits promote the progression of AD (Li et al., 2017). The extensive accumulation of immature autophagosomal vesicles has been observed in the AD brain, suggesting that autophagic proteolysis may be seriously compromised in this disease (Florez-McClure et al., 2007). The phenomenon of autophagosome accumulation is also noted in the Aβ transgenic *Caenorhabditis elegans* strain CL4176 (Florez-McClure et al., 2007).

Transgenic *C. elegans* expressing human Aβ have been used as AD model systems because of their short lifespan, well-characterized genome, and considerable homology with human genomes (Sorrentino et al., 2017). Various AD nematode models have been developed to investigate the molecular mechanism of Aβ toxicity and to screen therapeutic agents. These include GMC101, which expresses full-length Aβ_1–42_ in the body wall muscle (Sorrentino et al., 2017); CL4176, which is an effective model for screening AD drugs with potential therapeutic effects (Drake et al., 2003); and CL2355, which expresses Aβ in the neurons, and is used to observe Aβ-induced neuronal changes (Wu et al., 2006).

Based on the extremely complex pathogenesis of AD, the work of finding new therapeutic targets from pathology has become particularly important. We hope to explore the relationship between autophagy regulation and Aβ toxicity through transgenic nematode models. In the present study, we found that autophagy dysfunction caused an imbalance in protein homeostasis in Aβ transgenic strain GMC101. The intracellular expression of Aβ caused autophagosome accumulation, enhanced lysosomal activity, activated the rapamycin signaling pathway. Moreover, autophagy dysfunction resulted in oxidative stress and increased the accumulation of Aβ. While inhibiting the formation of autophagosomes delayed paralysis and had a neuroprotective effect. These results suggest the need to focus on the differential changes of autophagy during early stages of AD, and to maintain a stable autophagy flux while formulating AD treatment plans.

## Materials and Methods

### Strains and Maintenance Conditions

*Caenorhabditis elegans* strains CL4176 [(pAF29) myo-3p:Aβ1–42 + (pRF4) rol-6 (su1006)]; CL2355 [pCL45 (snb-1:Abeta 1-42:3′-UTR (long) + mtl-2:GFP]; GMC101 [unc-54p:A-beta-1-42:unc-54 3′-UTR + mtl-2p:GFP]; and CL2122 [(pPD30.38) unc-54 (vector) + (pCL26) mtl-2:GFP] were obtained from the Caenorhabditis Genetics Center (CGC; Minneapolis, MN, United States). If there is no special instructions, all strains are stored at 20°C. The worms were cultured on solid nematode growth medium (NGM) (Chen et al., 2020) plates containing a lawn of *Escherichia Coli* (*E. coli*) OP50.

The strains were routinely cultured and maintained at 16°C (for CL4176 and CL2355) on nematode growth medium (NGM) seeded with *Escherichia coli* OP50. From larval stage 1 (L1) or the young adult stage, they were fed with small molecule drugs 3-methyladenine (3-MA) (S2767, Selleck, China) or chloroquine (CQ) (S6999, Selleck, China) to interfere with autophagy flux. 3-MA is a selective phosphoinositide 3-kinase (PI3K) inhibitor that blocks autophagy through its action on phosphoinositide 3-phosphate kinase, and PI3K activity is necessary for the nucleation and assembly of membrane pools in the early stages of autophagosome formation (Zeng and Zhou, 2008). And 3-MA could provide neuroprotective effects in cerebral ischemia injury models. Chloroquine is a lysosomotropic weak base, which in the monoprotonated form diffuses into the lysosome, where it becomes diprotonated and becomes trapped. Protonated chloroquine then changes the lysosomal pH, thereby inhibiting autophagic degradation in the lysosomes. It is generally believed that CQ could deacidify lysosomes and inhibit autophagy (Palmisano et al., 2017).

### Construction of Transgenic Strains

Plasmid *lgg-1*p:mCherry:GFP:*lgg-1* (pMH878) was a gift from Professor Malene Hansen (Chang et al., 2017). Transgenic GMC101 or CL2122 strains expressing an extrachromosomal array were created by the gonadal microinjection of pMH878 (100 μg/ml) (Chang et al., 2017), which was subsequently integrated by γ-irradiation followed by outcrossing four times to GMC101 or CL2122 strains (Chang et al., 2017).

### RNA Sequencing (RNA-Seq)

GMC101 and CL2122 strains were synchronized (Fabian and Johnson, 1994), and their eggs allowed to hatch and develop to the young adult stage on NGM plates at 20°C. Then, the temperature was increased to 25°C to induce Aβ expression for 8 or 24 h. Gene expression in GMC101 and CL2122 strains was assessed by Novo Gene Corporation (Beijing, China). Sequencing libraries were generated using NEBNext^®^ Ultra™ RNA Library Prep Kit for Illumina^®^ (NEB, United States) following manufacturer’s recommendations and index codes were added to attribute sequences to each sample. In order to select cDNA fragments of preferentially 250∼300 bp in length, the library fragments were purified with AMPure XP system (Beckman Coulter, Beverly, United States). Then 3 μl USER Enzyme (NEB, United States) was used with size-selected, adaptor-ligated cDNA at 37°C for 15 min followed by 5 min at 95°C before PCR. Then PCR was performed with Phusion High-Fidelity DNA polymerase, Universal PCR primers and Index (X) Primer. At last, PCR products were purified (AMPure XP system) and library quality was assessed on the Agilent Bioanalyzer 2100 system. The clustering of the index-coded samples was performed on a cBot Cluster Generation System using TruSeq PE Cluster Kit v3-cBot-HS (Illumina) according to the manufacturer’s instructions. After cluster generation, the library preparations were sequenced on an Illumina NovaSeq platform and 150 bp paired-end reads were generated. Up- or down-regulated genes were identified by filtering RNA-seq data with the following cut-off: a twofold change in expression level and a false discovery rate analog of *p* < 0.05. KEGG pathway enrichment was applied using “clusterProfiler” with criteria *p* < 0.05 (Yu et al., 2012). All raw data and detailed experimental methods have been uploaded to GEO database, GEO number: GSE198684. Information on differential genes is in Supplementary Material.

### Quantitative Proteomics

GMC101 and CL2122 strains were synchronized, and their eggs allowed to hatch and develop to the young adult stage on NGM plates at 20°C. Then, the temperature was increased to 25°C to induce Aβ expression for 24 h. The label-free detection of protein expression in GMC101 and CL2122 strains was assessed by Novo Gene Corporation. A brief description is as follows: Peptides were separated in a home-made analytical column (15 cm × 150 μm, 1.9 μm), using a linear gradient elution. The separated peptides were analyzed by Q Exactive™ HF-X mass spectrometer (Thermo Fisher), with ion source of Nanospray Flex™(ESI), spray voltage of 2.1 kV and ion transport capillary temperature of 320°C. Full scan range from m/z 350 to 1,500 with resolution of 60,000 (at m/z 200), an automatic gain control (AGC) target value was 3 × 106 and a maximum ion injection time was 20 ms. The all resulting spectra were searched against database by the search engines: Proteome Discoverer 2.2 (PD 2.2, Thermo). Up- or down-regulated proteins were identified by filtering the data with the following cut-off: a twofold change in expression level and a false discovery rate analog of *p* < 0.05. Information on differential proteins is in Supplementary Material.

### Quantification of Autophagic Vesicles

*Caenorhabditis elegans* were mounted live on a 2% agarose pad in M9 medium containing 0.1% NaN_3_, and imaged using the Echo Revolve microscope at 40 ×. We selected the area above the pharynx of nematodes for statistics. The count method of mCherry:GFP:*lgg-1*-positive punctae was derived from the reported literature (Keith et al., 2016). Data were analyzed using one-way analysis of variance (ANOVA) or two-way ANOVA as applicable.

### Paralysis Assay

Aβ transgenic CL4176 nematodes were maintained on NGM at 16°C and synchronized (Zhang et al., 2016). They were treated with or without drugs at stage L1 for 36 h, then transferred to 23°C for transgene induction. This temperature shift stimulated Aβ expression, causing Aβ aggregation and leading to paralysis. Scoring was begun 27 h after the temperature shift, and the nematodes were considered to be paralyzed if they failed to move their bodies when touched and produced a “halo” of cleared bacterial lawn because they only moved their heads while feeding. We used PT_50_ as an index (the time interval from the onset of paralysis at which 50% of the nematodes were paralyzed). For example, a PT_50_ of 4.1 h for the control was obtained by subtracting the onset time of paralysis, 29 h, from the time when 50% of the nematodes were paralyzed (33.1 h). The assay was performed at least three times. And each individual group contained more than 30 nematodes. Statistical analysis was conducted with GraphPad Prism 6.0 software, and *p* values were calculated using the log-rank test.

### LysoTracker Red Staining

The acidophilic dye LysoTracker Red (C1046; Beyotime Biotechnology, Shanghai, China) (Imanikia et al., 2019) was used at a final concentration of 15 μM. Synchronized GMC101 or CL2122 strains maintained at 20°C were treated with LysoTracker at stage L1 until the young adult stage, then transferred to 25°C for transgene induction. After 24 h, nematodes were washed twice in fresh M9 (without LysoTracker) and imaged using a Revolve microscope (Imanikia et al., 2019).

### Gene Expression Analysis by Quantitative PCR

Total RNA was extracted using Trizol A + (Tiangen, Beijing, China) and reverse-transcribed into cDNA. Expressed genes were amplified in triplicate and quantified by PCR using a SYBR Green PCR Mix (B21702, Bimake, China) with the Roche LightCycler system. Data was analyzed using the 2^–ΔΔCT^ method. The primer sequences used for quantitative PCR (qPCR) are provided in Supplementary Material 1.

### RNA Interference

RNA interference (RNAi) experiments were based on a reported protocol (Li et al., 2018). In brief, RNA was delivered to nematodes by feeding, so gravid CL4176 adults were bleached, and eggs were placed on NGM dishes containing 1 mM isopropyl β-D-1-thiogalactopyranoside (IPTG). An HT-115 (DE3) bacterial colony containing L4440 or the target gene plasmid was inoculated in LB broth containing 100 μg/mL ampicillin and 12.5 μg/mL tetracyclines, and grown for 8 h in a 37°C shaker. The bacteria were plated on the NGM dish containing IPTG 1 h prior to the addition of nematodes. The synchronized CL4176 strain was grown on RNAi NGM plates from eggs to adults for two generations. Synchronized L1 worms were treated with or without 3-MA or CQ for the paralysis assay as described above (Zhang et al., 2016).

### Measurement of Reactive Oxygen Species

Endogenous reactive oxygen species (ROS) levels were measured using 2′,7′-dichlorofluorescein diacetate (H2DCF–DA), which reacts with endogenous ROS to generate a fluorescent product (Yang et al., 2018). Nematodes were treated as in the paralysis assay, and ROS was measured at the point of paralysis. They were then incubated with 50 μM H2DCF–DA for 30 min at 37°C, and the fluorescence intensity was measured at excitation and emission wavelengths of 485 and 535 nm, respectively. The assay was performed in triplicate.

### Western Blotting

The GMC101 strain was synchronized, and eggs were allowed to hatch and develop to the L4 stage on NGM plates with or without 3-MA or CQ at 20°C. Then, the temperature was increased to 25°C and maintained for 24 h. Nematodes were collected from the plates with M9 buffer and washed twice to eliminate bacteria. Samples were heated at 100°C in sample loading buffer for 10 min and centrifuged at 10,000 g for 10 min (Sangha et al., 2015). Collected supernatant was boiled with loading buffer at 100°C for 5 min before being loaded into the gel. A 10–180 kDa protein marker (PR1910, Solarbio, China) was used as an indicator of molecular weight (Zhu et al., 2019). Samples were run at 40 V for 40 min on a stacking gel, and at 80 V for 120 min on a separating gel. The gel was then transferred to a polyvinylidene fluoride membrane using 20% methanol transfer buffer at 100 V for 1 h. Blots were blocked in Tris-buffered saline with Tween 20 + 5% skimmed milk for 1 h. The Aβ protein levels were detected with 6E10 monoclonal antibody (dilution 1:500; 803014, BioLegend), with an anti-β-actin antibody (dilution 1:2,000; 60008, Proteintech) as a control. mCherry -GFP-LGG-1 was detected with a primary antibody against LGG-1 (dilution 1:1,000; Cell Signaling Technology). The horseradish peroxidase-conjugated goat anti-mouse antibody (1:2,000, Cell Signaling Technology) was used as the secondary antibody. Incubate the primary antibody overnight, and incubate the secondary antibody for 2 h at room temperature. Images were captured using a Chemiscope 3400 mini western blot imaging system (Amersham Imager 600, GE, United States). Mean densities of the Aβ bands were analyzed using Image J software.

### Chemotaxis Assays

The chemotaxis response in *C. elegans* is mediated by the activation of several sensory neurons and interneurons to stimulate motor neurons. Chemotaxis assays were performed as described previously (Wu et al., 2006). Synchronized transgenic *C. elegans* CL2355 and its control strain CL2122 were treated with or without 3-MA or CQ starting from L1 stage. They were cultured at 16°C for 36 h, then at 23°C for another 36 h, then collected and assayed in 100 mm plates. A total of 1 μl 0.25 M sodium azide and 1 μl odorant (0.5 M sodium acetate in 100% ethanol) were added to the “attractant” spot. On the opposite side of the attractant spot, 1 μl control odorant (100% ethanol) and 1 μl sodium azide were added. Immediately afterward, 2 μl nematodes (n = approximately 60) were pipetted into the center of the plate, incubated at 23°C for 1 h, and the number of nematodes in each quadrant was scored. The chemotaxis index (CI) is a measure of the fraction of worms that move to the location of the attractant, and was calculated as follows:


CI=⁢(number⁢of⁢worms⁢in⁢both⁢attractant⁢quadrants-number⁢of⁢worms⁢in⁢both⁢control⁢quadrants)/total⁢number⁢of⁢scored⁢worms.

### 5-Hydroxytryptamine Sensitivity Assay

CL2355 worms were egg-synchronized and placed onto fresh NGM plates seeded with OP50, treated with or without 3-MA or CQ at 16°C for 36 h, then transferred to 23°C for 36 h. They were collected with M9 buffer, and the number of paralyzed worms after exposure to 5 mg/mL 5-hydroxytryptamine (5-HT) in a 96-well plate for 24 h was counted (Wu et al., 2006). The assay was performed at least three times.

### Statistical Analysis

GraphPad Prism 6.0 software was used for statistical analyses. For paralysis assays, *p*-values were calculated using the log-rank test. The Student’s *t*-test was used to compare two groups. One-way ANOVA with Duncan’s test was performed to compare multiple groups. *p* < 0.05 was considered statistically significant.

## Results

### Human Aβ Expression Results in Autophagosome Accumulation in *Caenorhabditis elegans* Muscle

**FIGURE 1:**
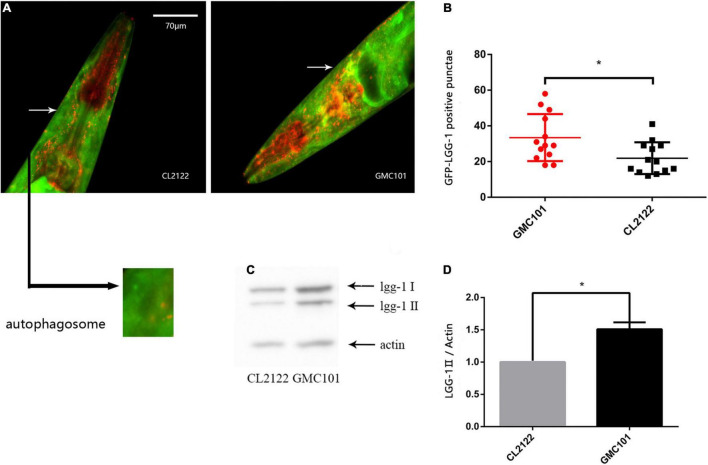
Autophagosome accumulation in GMC101 and CL2122 strains. **(A)** Punctae formation as shown by fluorescence microscopy. **(B)** Scoring of punctae showing a significant increase in the GMC101 strain (*n* = 15 for CL2122 and *n* = 18 for GMC101, *p < 0.05 by the Student’s *t*-test). **(C)** Representative western blot of mCherry::GFP::lgg-1 in CL2122 and GMC101 strains. **(D)** Quantified western blot gel intensities, as determined by ImageJ software (*n* = 3, *p < 0.05 by the Student’s *t*-test).

The accumulation of autophagic vacuoles at various stages of maturation has been reported in the CL4176 strain (Florez-McClure et al., 2007). In order to further confirm the phenomenon is widespread in transgenic Aβ strain, we constructed a dual-fluorescent mCherry:GFP:LGG-1 protein system using the GMC101(Aβ_1–42_) strain and CL2122(control) strain to monitor autophagosomes by microinjection (Chang et al., 2017). With this reporter, autophagosomes are visualized as both GFP- and mCherry-positive punctae. By counting the number of GFP-LGG-1 positive punctae, we observed clear autophagosome accumulation in the GMC101 strain compared with the CL2122 strain (Figures 1A,B). To further confirm this, we used western blotting to measure the expression of lgg-1-II, which binds to autophagosomes (Springhorn and Hoppe, 2019). The protein level was significantly increased in the GMC101 strain compared with the CL2122 strain (Figures 1C,D), which was supportive of autophagosome accumulation in the Aβ transgenic strain.

### Autophagosome Accumulation in GMC101 Is Not Due to Down-Regulated Lysosomal Acidity but Due to Reactive Oxygen Species-Mediated Abnormal Autophagosome Fusion

**FIGURE 2:**
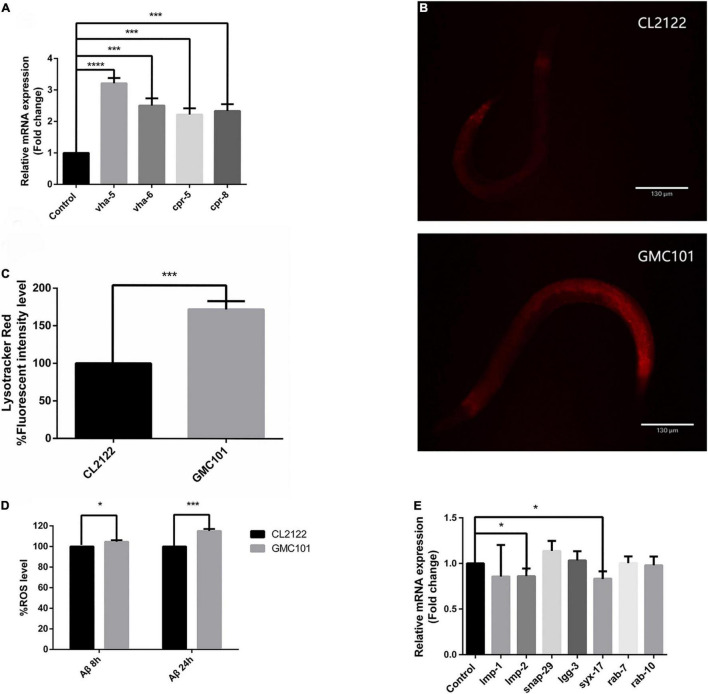
Aβ enhanced lysosomal acidity in GMC101 compared with CL2122 strains. **(A)** V-ATPase and cathepsin mRNA expression. **(B)** Representative fluorescence images of CL2122 and GMC101 strains stained with LysoTracker Red after Aβ induction for 24 h. **(C)** Quantified CL2122 and GMC101 fluorescence intensities (*n* = 10 for CL2122 and *n* = 11 for GMC101, repeated three times. ***p < 0.001 by the Student’s *t*-test). **(D)** Levels of ROS in transgenic *C. elegans*. **(E)** Autophagosome and lysosome fusion gene mRNA expression following Aβ induction for 24 h. Each group contained about 60 worms (*n* = 3, *p < 0.05; ***p < 0.001 by the Student’s *t*-test; ****p < 0.0001).

Decreased lysosomal acidity caused by aging has been identified as causative of abnormal autophagic flux in the brain of AD patients (Zare-Shahabadi et al., 2015). We speculated that autophagosome accumulation in the GMC101 strain is also associated with decreased lysosome acidity. To investigate this, we first measured mRNA expression levels of V-ATPase and cathepsin genes, which maintain lysosomal acidity (Ernstrom et al., 2012) and degrade Aβ (Hook et al., 2020), respectively. However, contrary to our expectations, the transcription of V-ATPase and cathepsin genes was significantly increased 24 h Aβ post-induction (Figure 2A and Table 1). To better observe Aβ-induced changes in lysosomal acidity, we carried out LysoTracker Red staining, and detected significantly increased levels of staining in the GMC101 strain compared with the CL2122 strain (Figures 2B,C). These results indicate that lysosomal acidity is enhanced in the GMC101 strain, suggesting its autophagosome accumulation is not caused by decreased lysosome acidity. In fact, Aβ induction appears to not only enhance the expression of lysosome-related genes but also to increase lysosomal acidity.

**TABLE 1:** Differential gene expression verified by qRCR.

Gene ID	Fold Change (RNA-seq)	Fold Change (qPCR) ± SEM
vha-5	3.5933	3.210 ± 0.0981
vha-6	2.0691	2.507 ± 0.1317
cpr-5	2.4064	2.217 ± 0.1172
cpr-8	2.3219	2.333 ± 0.1241
atg-16.2	0.3769	0.776 ± 0.0058
epg-8	0.4476	0.672 ± 0.0203
lgg-1	0.4691	0.663 ± 0.0412
atg-4.2	0.3542	0.879 ± 0.0057
atg-18	0.4426	0.732 ± 0.0225
hsp-16.2	33.1284	8.002 ± 1.710
hsp-70	15.6707	8.716 ± 0.755

*The experimental method and the number of repetitions have been stated in the method. The primer sequence is in Supplementary Material 1.*

It was reported that Aβ induction could increase ROS levels in the transgenic APP mouse model (3×Tg-AD) (Ghosh et al., 2012), while ROS was shown to cause autophagosome accumulation (Wang et al., 2019). We evaluated the nematode redox status by measuring intracellular ROS levels with H_2_DCF-DA (Figure 2D). The transcription levels of autophagosome–lysosomal fusion genes were also measured (Figure 2E). Following Aβ induction, ROS levels increased while fusion-related gene transcription was down-regulated. This suggests that ROS prevented the fusion of autophagosomes and lysosomes.

### Reducing Autophagosome Accumulation Delays Aβ-Induced Paralysis and Suppresses Neuronal Aβ Expression-Induced Defects in Chemotaxis Behavior and 5-Hydroxytryptamine Sensitivity

**FIGURE 3:**
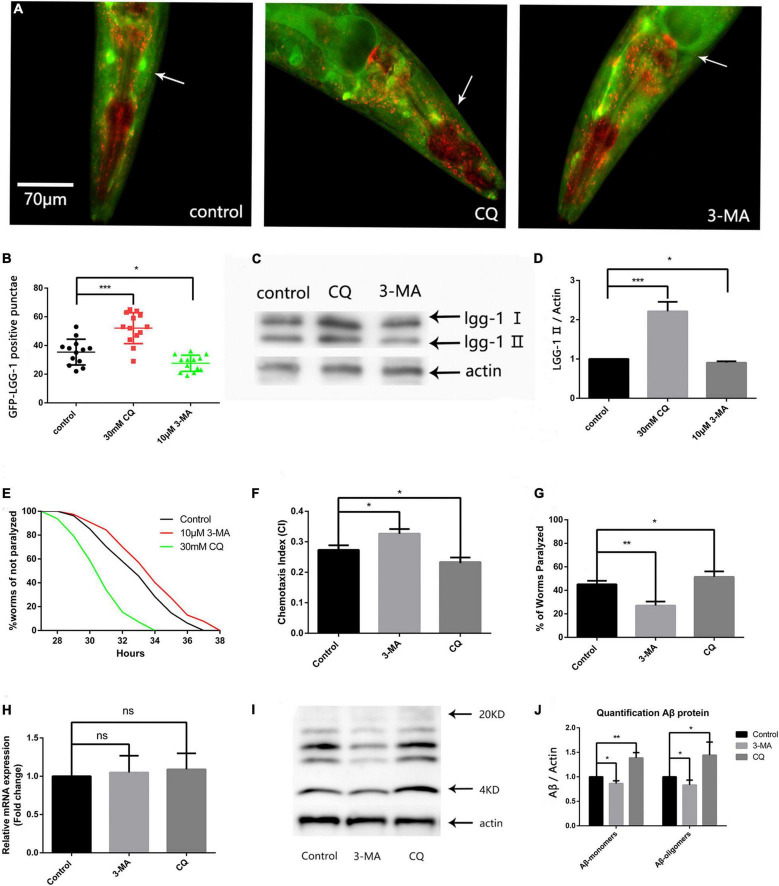
Reducing autophagosome accumulation delays Aβ-induced paralysis in the CL4176 strain and suppresses neuronal Aβ expression-induced defects in chemotaxis behavior and 5-HT sensitivity. **(A)** Punctae formation in the GMC101 strain as shown by fluorescence microscopy. **(B)** Scoring of punctae (*n* = 13 per group, *p < 0.05; ***p < 0.001 by the Student’s *t*-test). **(C)** Representative western blot of mCherry::GFP::lgg-1. **(D)** Quantified western blot gel intensities, as determined by ImageJ software (*n* = 3, *p < 0.05; ***p < 0.001 by the Student’s *t*-test). **(E)** 3-MA delayed Aβ-induced paralysis and CQ accelerated Aβ-induced paralysis in the CL4176 strain (*n* = 3, **p < 0.01; ***p < 0.001 by the log-rank test). **(F)** 3-MA significantly improved chemotaxis while CQ significantly impaired chemotaxis. **(G)** Serotonin hypersensitivity was restored to normal levels by 3-MA. CQ worsened serotonin hypersensitivity. Each group contained about 60 worms (*n* = 3, *p < 0.05; **p < 0.01). **(H)** Aβ mRNA expression in treated and untreated nematodes. **(I)** Representative western blot of Aβ species in transgenic nematodes. **(J)** Quantified western blot gel intensities, as determined by ImageJ software (*n* = 3, *p < 0.05; **p < 0.01 by the Student’s *t*-test).

Because abnormally high ROS levels caused autophagosome accumulation (Wang et al., 2019), and abnormal organelles destroyed protein homeostasis, we next explored the effect of autophagosome accumulation on protein homeostasis. First, we used the fluorescent nematode mentioned in the above article to evaluate the effect of 3-MA and CQ on the number of GFP-LGG-1 positive punctae. Both fluorescence analysis (Figures 3A,B) and western blotting (Figures 3C,D) showed that 3-MA significantly reduced, while CQ aggravated, autophagosome accumulation.

Then we used the CL4176 model to evaluate the effect of autophagy intervention on protein homeostasis. Our results showed that 10 μM 3-MA caused a delay in Aβ-induced paralysis, and that the PT_50_ value was increased by 30.54% compared with the control (Figure 3E and Table 2). This suggests that inhibiting the formation and reducing the accumulation of autophagosomes has a protective effect against AD in nematodes. Conversely, 30 mM CQ significantly accelerated Aβ-induced paralysis and decreased the PT_50_ value by 37.84% (Figure 3E and Table 2), showing the clear impact of lysosome deacidification on protein homeostasis. These results are consistent with the observed decrease in lysosomal activity mediated by aging responsible for autophagy dysfunction (Lipinski et al., 2010), which promotes the progression of AD.

**TABLE 2:** Paralysis assay in CL4176 strain nematodes.

	N	PT_50_	*P*-value	Extension percentage (%)
Control	3	3.70 ± 0.10		
10 μM 3-MA	3	4.83 ± 0.20	0.007**	30.54
30 mM CQ	3	2.30 ± 0.06	0.0003***	−37.84
l4440	3	2.46 ± 0.14		
epg-8 RNAi	3	3.10 ± 0.05	0.0155*	26.01
epg-8 RNAi CQ	3	2.06 ± 0.26	ns	−16.26
l4440	3	4.66 ± 0.15		
hsp-16.2 RNAi	3	3.97 ± 0.18	0.0375*	−14.99
hsp-16.2 RNAi 3-MA	3	3.36 ± 0.40	0.0392*	−27.80
l4440	3	4.16 ± 0.03		
hsp-70 RNAi	3	3.56 ± 0.33	0.0002***	−14.00
hsp-70 RNAi 3-MA	3	2.86 ± 0.12	0.0005***	−31.19

The experimental method and the number of repetitions have been stated in the method. *p < 0.05; **p < 0.01; ***p < 0.001.

Because limiting autophagosome accumulation delayed paralysis in the CL4176 strain, we next explored whether reducing autophagy accumulation had neuroprotective effects by characterizing the neuronal controlled behaviors of chemotaxis and 5-HT sensitivity in the CL2355 strain, in which Aβ is expressed in neuronal cells (Wu et al., 2006). The CI is a measure of the fraction of worms that are able to arrive at the location of the attractants. 5-HT is a key neurotransmitter that modulates several behaviors of *C. elegans*. When exogenous 5-HT is applied to the nematodes, they become paralyzed as a result of the sensitivity to excessive 5-HT (Wu et al., 2006).

Figure 3F shows that 10 μM 3-MA significantly improved the CI compared with the untreated control (CI_control_, 0.2733 ± 0.0088 vs. CI_3–MA_, 0.3300 ± 0.0057, *n* = 3, **p < 0.01), suggesting that 3-MA has a significant neuroprotective effect in nematodes. We also found that 30 mM CQ significantly reduced the CI compared with the control (CI_control_, 0.2733 ± 0.0088 vs. CI_CQ_, 0.2333 ± 0.0088, *n* = 3, *p < 0.05).

Using the ability of nematodes to take up exogenous 5-HT, we next evaluated the effects of 3-MA and CQ on the nematode nervous system. Figure 3G shows a percentage paralysis of 3-MA-treated worms of 27.00 ± 1.98% and CQ-treated worms of 53.23 ± 1.19%, compared with the control of 45.10 ± 1.75%. Therefore, 10 μM 3-MA ameliorated the phenotypic defect in the CL2355 strain, while it was made worse by 30 mM CQ. Thus, the inhibition of autophagosome formation by 3-MA exerted a significant neuroprotective effect in the nematode model of Aβ-induced neurotoxicity, while abnormal autophagosome degradation exacerbated Aβ-induced neurotoxicity.

To further evaluate protein homeostasis in nematodes, we measured the level of Aβ mRNA by qPCR, but found no significant difference between 3-MA-treated and CQ-treated nematodes (Figure 3H). However, western blotting of Aβ protein levels (Sangha et al., 2015) showed that 30 mM CQ significantly increased the expression of Aβ monomers (fold-change: 1.387 ± 0.062, *n* = 3, **p < 0.01) and oligomers (fold-change: 1.443 ± 0.153, *n* = 3, *p < 0.05) (Figure 3I). Moreover, 10 μM 3-MA significantly reduced the expression of Aβ monomers (fold-change: 0.863 ± 0.031, *n* = 3, *p < 0.05) and oligomers (fold-change: 0.833 ± 0.057, *n* = 3, *p < 0.05) (Figure 3J). This phenomenon may be associated with the alleviation of autophagosome accumulation, and requires further exploration.

### Autophagosome Reduction by the PI3K Complex Delays Aβ-Induced Paralysis in CL4176 Nematodes and Has a Neuroprotective Effect

**FIGURE 4:**
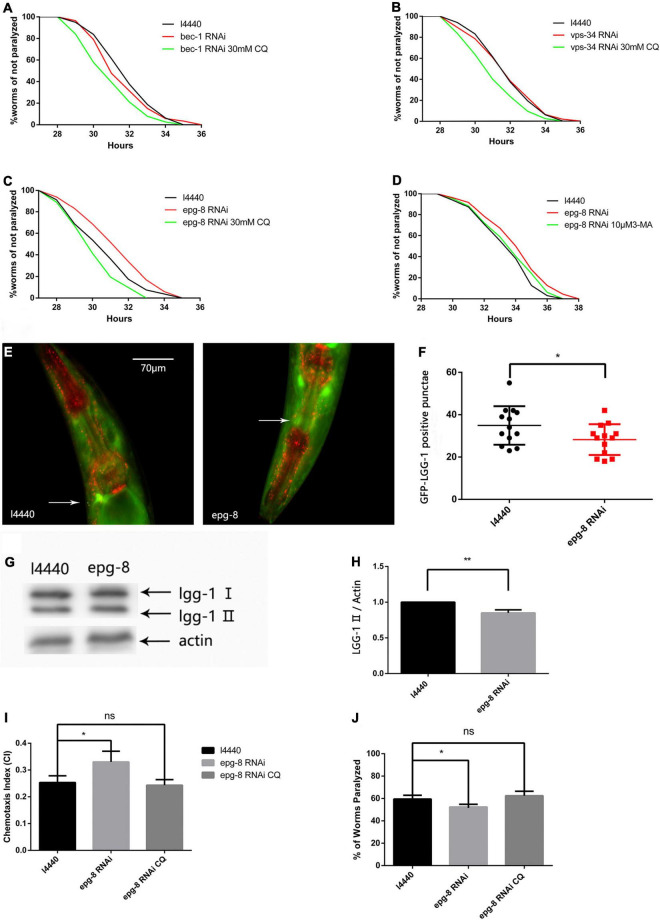
The CL4176 strain was fed either vector l4440 control bacteria or bacteria expressing RNAi for *bec-1* **(A)**, *vps-34* **(B)**, or *epg-8* **(C,D)**. RNAi of *epg-8* delayed Aβ-induced paralysis which could be offset by CQ. (*n* = 3, *p < 0.05 by the log-rank test). Each individual group contains more than 30 nematodes. **(E)** Punctae formation in the GMC101 strain with or without RNAi bacteria as shown by fluorescence microscopy. **(F)** Scoring of punctae with or without RNAi bacteria (*n* = 13 per group, *p < 0.05 by the Student’s *t*-test). **(G)** Representative western blot of mCherry::GFP::lgg-1. **(H)** Quantified western blot gel intensities, as determined by ImageJ software (*n* = 3, **p < 0.01 by the Student’s *t*-test). **(I)** Chemotaxis of the CL2355 strain fed either vector l4440 control bacteria or bacteria expressing RNAi for *epg-8*. **(J)** Serotonin hypersensitivity was restored by *epg-8* RNAi and reversed by CQ. Each group contains about 60 worms (*n* = 3, *p < 0.05).

Because the PI3K inhibitor 3-MA delayed paralysis, we next determined whether suppressing PI3K gene transcription would have the same effect. We used RNAi to decrease the expression of PI3K complex-related genes (Figures 4A–C) *bec-1*, *vps-34*, and *epg-8* (Zhang and Baehrecke, 2015). Figure 4C shows that *epg-8* RNAi delayed Aβ-induced paralysis, and increased the PT_50_ value by 26.01% compared with the control (Table 2). While *bec-1* or *vps-34* RNAi did not prolong paralysis. Then we performed RNAi on the fluorescent model. Autophagosome accumulation was significantly reduced (Figures 4E–H) when we reduced the expression of *epg-8* by RNAi, indicating that reducing autophagosome accumulation alleviates Aβ-induced toxicity. No significant differences were observed after intervention on the other two genes. CI and 5-HT sensitivity results also provided strong support for this (Figures 4I,J), with *epg-8* RNAi significantly increasing the CI compared with the control (CI_l4440_, 0.2533 ± 0.0145 vs. CI_epg–8RNAi_, 0.3300 ± 0.0231, *n* = 3, *p < 0.05). Moreover, *epg-8* RNAi worms had a percentage paralysis of 59.43 ± 1.99% in the 5-HT sensitivity assay compared with 54.40 ± 1.36% for the l4440 control, indicating that serotonin hypersensitivity was restored to normal levels by *epg-8* RNAi. This protective effect was offset by CQ (Figures 4C,I,J). Thus, stopping the formation of autophagosomes and reducing their accumulation decreased Aβ-induced neural damage. 3-MA administration had no additive effects on the basis of *epg-8* RNAi (Figure 4D).

### The Neuroprotective Effect of Inhibiting Autophagosome Accumulation Requires the Participation of Small Molecular Chaperones

**FIGURE 5:**
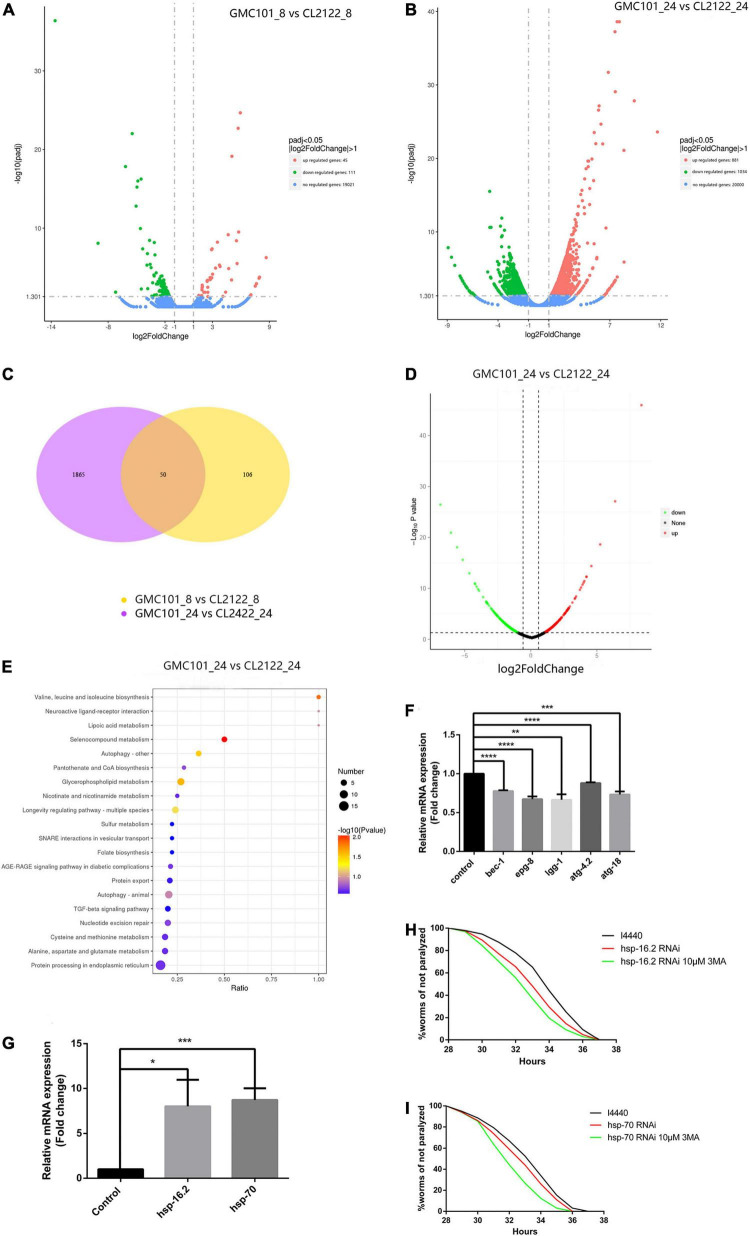
Molecular chaperones are essential for Aβ toxicity in transgenic nematodes. **(A)** After 8 h Aβ induction, 45 genes were up-regulated and 111 down-regulated in GMC101 compared with CL2122 strains. **(B)** After 24 h Aβ induction, 881 genes were up-regulated and 1034 down-regulated in GMC101 compared with CL2122 strains. **(C)** Venn diagram showing the overlap among genes showing significant differential expression (*p* < 0.05) between samples. **(D)** After 24 h Aβ induction, 273 proteins were up-regulated and 301 down-regulated in GMC101 compared with CL2122 strains. **(E)** KEGG pathway enrichment of differentially expressed proteins between CL2122 and GMC101 strains. x and y axes represent GeneRatio and enriched KEGG pathway, respectively. Color represents enrichment significance, and bubble size represents gene count. **(F)** Validation of differentially expressed genes screened by RNA-seq (*n* = 3, **p < 0.01; ***p < 0.001; ****p < 0.0001 by the Student’s *t*-test). **(G)** Validation of differentially expressed genes screened by RNA-seq. The CL4176 strain was fed either vector l4440 control bacteria or bacteria expressing RNAi for *hsp-16.2* **(H)** and *hsp-70* **(I)**. RNAi for molecular chaperones accelerated Aβ-induced paralysis in the CL4176 strain (*n* = 3, *p < 0.05 by the log-rank test).

To better understand the Aβ-induced mechanism of autophagy dysfunction, we performed RNA-seq and proteomic analysis. RNA-seq identified 45 genes that were up-regulated and 111 that were down-regulated in the GMC101 strain compared with the CL2122 control after 8 h Aβ induction, as well as 881 genes that were up-regulated and 1,034 down-regulated after 24 h induction (Figures 5A–C). By enriching the differentially expressed genes associated with autophagy, we identified those with significant differences (Tables 3, 4). The results of qPCR supported RNA-seq findings (Figure 5F and Table 1), and showed that autophagy-related gene expression trends were inconsistent which implies that complex changes occur in the autophagy pathway during Aβ induction.

**TABLE 3:** Autophagy-related gene expression after 8 h Aβ induction.

Wormbase ID	Gene name	Log_2_(Foldchange)
WBGene00002015	hsp-16.1	10.73201484
WBGene00002019	hsp-16.48	9.993542326
WBGene00002020	hsp-16.49	9.414459887
WBGene00002026	hsp-70	8.695174305
WBGene00002016	hsp-16.2	7.349540251
WBGene00002018	hsp-16.41	7.128486268
WBGene00002017	hsp-16.11	6.349008531
WBGene00002021	hsp-17	1.62722773
WBGene00002008	hsp-4	1.491200843
WBGene00000785	cpr-5	1.427380633
WBGene00002007	hsp-3	1.303493789
WBGene00002011	hsp-12.2	1.280543166
WBGene00006921	vha-12	1.094806355
WBGene00008427	atg-10	−1.583421545

**TABLE 4:** Autophagy-related gene expression after 24 h Aβ induction.

Wormbase ID	Gene name	Log_2_(Foldchange)
WBGene00002026	hsp-70	3.976840027
WBGene00011906	hsp-12.1	2.032493207
WBGene00006914	vha-5	1.845318311
WBGene00000785	cpr-5	1.266866182
WBGene00021070	cpr-8	1.215308479
WBGene00002023	hsp-25	1.107285903
WBGene00002024	hsp-43	1.111466983
WBGene00006915	vha-6	1.049060101
WBGene00008427	atg-10	−2.831118741
WBGene00002982	lgg-3	−1.63575482
WBGene00014080	atg-4.2	−1.497132712
WBGene00019427	atg-16.2	−1.407504423
WBGene00017178	atg-16.1	−1.272882712
WBGene00018294	atg-18	−1.175782797
WBGene00013695	epg-8	−1.159589162
WBGene00010882	atg-7	−1.124701339
WBGene00002980	lgg-1	−1.092218465
WBGene00021922	atg-3	−1.046291041

Proteomic data showed that 273 proteins were up-regulated and 301 were down-regulated in the GMC101 strain compared with the CL2122 control after Aβ induction (Figure 5D). After using KEGG, we found that autophagy signaling pathways were enriched as differential signaling pathways between CL2122 and GMC101 strains (Figure 5E). Among the top 50 up-regulated proteins, we found that let-363 protein (mammalian mTOR) was significantly up-regulated. This also explained why the transcription level of autophagosome formation genes is down-regulated. These results indicated that Aβ induction impacts on the autophagy signaling pathway and generated a series of downstream events.

To explore the neuroprotective effect of 3-MA, we again conducted RNA-seq (data not shown) and identified 70 KEGG pathways that were enriched with differentially expressed genes between GMC101 and 3-MA groups. Significantly enriched pathways included the Wnt signaling pathway, transforming growth factor-beta signaling pathway, ubiquitin-mediated proteolysis, and protein processing in the endoplasmic reticulum, suggesting that inhibiting autophagosome formation activates pathways that maintain protein homeostasis.

Autophagy and molecular chaperones were previously shown to have compensatory phenomena in the degradation of misfolded proteins (Park and Cuervo, 2013). Here, we observed a significant increase in the expression of molecular chaperone genes after Aβ induction by RNA-seq (Tables 3, 4). These findings were verified by qPCR (Figure 5G and Table 1), suggesting that the protective effect of 3-MA requires molecular chaperones. We performed RNAi on the small molecule chaperones *hsp16.2* and *hsp-70* (Figures 5H,I), which reversed the effect of 3-MA in delaying the paralysis time (Table 2). This strongly indicates that the effect of 3-MA in prolonging nematode paralysis depends on the participation of small molecular chaperones.

## Discussion

Although AD is the most common neurodegenerative disease, its pathogenesis remains unclear (Lane et al., 2018). Here, we used the *C. elegans* AD model to study autophagy dysfunction as a pathological phenomenon following the transgenic expression of human Aβ. We verified that protein homeostasis was disrupted and that autophagosome accumulation occurred following the induction of Aβ expression in *C. elegans* (Figure 1). Autophagosome accumulation in the brain was previously shown to mainly result from reduced lysosomal activity (Sarkis et al., 1988), dynein transport disorders (Lee et al., 2011), and abnormal fusion (Yang et al., 2019).

In our study, we first evaluated the activity of lysosomes regulated by aging (Cairo and Villarroya, 2020) or Aβ (Song et al., 2020) in AD. However, we found that Aβ induction enhanced nematode lysosome activity (Figure 2B), which manifested as significant changes in the transcription of V-ATPase and cathepsin genes (Figure 2A and Table 1). V-ATPase is responsible for transferring H^+^ from the cytoplasm to the lysosome (Ernstrom et al., 2012), while cathepsin B and cathepsin D are thought to degrade Aβ (Hook et al., 2020). The observed enhancement of lysosomal activity and activated cathepsin transcription suggested that lysosomes are highly sensitive to misfolded proteins under non-aging conditions. However, lysosomal activity is continuously reduced during aging, and the Aβ degradation ability is also weakened, leading to further aggravation of abnormal autophagy. We used CQ to mimic autophagy deterioration through aging, which confirmed our speculation (Figures 3E–G), and showed that lysosomes are key organelles for maintaining protein homeostasis.

After ruling out lysosome inactivation as causative of autophagosome accumulation, we explored other influencing factors. Dynein transport was also excluded in our nematode model (muscle cells are morphologically different from nerve cells, and lysosomal transport is less dependent on dynein), suggesting that autophagosome accumulation was most likely caused by abnormal lysosome fusion. This was confirmed by our ROS measurements (Figure 2D) and detection of fusion gene transcription (Figure 2E). Both the perforin effect of Aβ (Julien et al., 2018) and Aβ-induced oxidative stress can lead to abnormal fusion. However, the exact mechanism is still unclear, and requires further study.

3-MA was reported to have therapeutic effects in cerebral ischemia injury models by reducing autophagosome accumulation (Wang and Wu, 2020). We observed that 10 μM 3-MA prolonged the paralysis time in the CL4176 strain and had a neuroprotective effect in the Aβ transgenic nematode model (Figures 3E–G). Reducing autophagosome formation by RNAi had the same effect (Figure 4), suggesting that an imbalance of protein homeostasis caused by abnormal autophagy could be relieved by inhibiting autophagosome formation. On the one hand, reducing the number of abnormal organelles alleviates the stress level, on the other hand it may activate the UPS signaling pathway to improve intracellular pressure. Therefore, maintaining basic levels of autophagy is important in AD treatment.

Molecular chaperones were previously shown to be up-regulated following Aβ expression in an AD model (Lackie et al., 2017). Moreover, wild-type Aβ, but not an Aβ single chain dimer, could be sequestered in HSP-16.2-containing inclusions (Ai et al., 2018), suggesting the existence of a conformation-dependent interaction between chaperone and Aβ *in vivo*. We detected significant changes in small molecular chaperone expression at both the transcription and protein levels following Aβ induction (Table 1). Additionally, the paralysis rate was significantly increased when we used RNAi to decrease heat shock protein gene transcription in the CL4176 strain (Figures 5H,I), indicating that small molecular chaperones play a key role in maintaining protein homeostasis. It is reported that there is a compensatory mechanism between the ways to maintain protein homeostasis (Ji and Kwon, 2017). We hypothesize that 3-MA reduces autophagosome formation and has a protective effect that is molecular chaperone-dependent. RNAi on the small molecule chaperones hsp16.2 and hsp-70 reversed the neuroprotective effect of 3-MA, indicating that it requires the participation of chaperones.

Autophagy is a double-edged sword in AD (Yin et al., 2017), because enhanced autophagy is thought to have therapeutic effects (Friedman et al., 2015), but the accumulation of autophagosomes in the brain can cause serious oxidative stress and trigger apoptosis. Taken together, our findings show that maintaining normal levels of autophagy and lysosomal activity and reducing autophagosome accumulation could relieve Aβ-induced injuries. Further study is needed to explore the relationship between protein homeostasis and Aβ, which is essential for the treatment of AD.

## Data Availability Statement

The datasets presented in this study can be found in online repositories. The names of the repository/repositories and accession number(s) can be found below: https://www.ncbi.nlm.nih.gov/geo/, GSE198684.

## Author Contributions

HL, HW, and XC conceived and designed the experiments. HL, YG, and CZ performed the experiments. HL, BM, and MW analyzed the data. HL, HW, and XC wrote the manuscript. All authors read and approved the final manuscript.

## Conflict of Interest

The authors declare that the research was conducted in the absence of any commercial or financial relationships that could be construed as a potential conflict of interest.

## Publisher’s Note

All claims expressed in this article are solely those of the authors and do not necessarily represent those of their affiliated organizations, or those of the publisher, the editors and the reviewers. Any product that may be evaluated in this article, or claim that may be made by its manufacturer, is not guaranteed or endorsed by the publisher.

## Supplementary Material

Supplementary Table 1 The 8 h RNA-seq differential gene data.

Supplementary Table 2 The 24 h differential gene data.

Supplementary Table 3 The proteomic differential protein data.

Supplementary Data Sheet 1 The primer list.
