# Supplementary material for: Autophagy Regulation Influences β-Amyloid Toxicity in Transgenic Caenorhabditis elegans
Source: Front Aging Neurosci. 2022 May 12;14:885145. doi: 10.3389/fnagi.2022.885145 (PMC9133694; doi:10.3389/fnagi.2022.885145)
Supplement: Supplementary Data Sheet 1 — The primer list. [file Data_Sheet_1.PDF]

Primer list:

vha-5 F:CTTCATGGAAACGCGACTGT R:CGGTAACGAACACCATGTGC

vha-6 F:CTTGCTCACGCACAGCTTTC R:TGAAGGAAGGCGGAAAGACC

cpr-5 F:CTCCGACGCTATTCCAGACC R:GCGTAGGCGGTAGATCCAAA

cpr-8 F:TTTCCTGTGGGGAAGGGTGT R:TGACCCTGTTTGTTGCCAGT

lmp-1 F:GTCAGTTGGGAAAACGGTGC R:CGCTGCTTTTGAAGCCTTGT

lmp-2 F:CGCTGCTTTTGAAGCCTTGT R:AGTGTCCGCTGTTGAGGATG

snap-29 F:ACCACGCAAATGACACAACG R:TTTGGATTGCGGGACAGTCG

lgg-3 F:ACACCACGTTTGAGACGCTA R:GTAGGCCGGTGTAATGCTGT

syx-17 F:CAAGATCCCTACCCGCTGTC R:GCATAAAGTGGAGCCTTCGC

rab-7 F:TTCCTCACACGCGACGTAAA R:CGGCTCCACGATAAAAAGCG

rab-10 F:AGCTTCCACGAGACTTCAGC R: CCTCTGTGGTTGCACTGGAT

bec-1 F:TGTTGAAAGAGCTCAAGGATCG R:GGGGAAAAGGCAGAATTCCAG

epg-8 F:GCGGTAAACGCTACACAAAGA R:CCATCCGCTGAGATTCCTGG

lgg-1 F: CGTGCCGAAGGAGACAAGAT R:CTTCCTCGTGATGGTCCTGG

atg-4.2 F:TCCGGGTGGCAATCTTTCAT R:CGAGCGAAGACCACTTTCATC

atg-18 F:AAGTTGGGGAGCTGATGACG R:TGGTCTAAACGGATATGCTTGCT

hsp-16.2 F:CCATCTGAGTCTTCTGAGATTGTTA R:TTCAAGTTTATTGCAGCGAACA

hsp-70 F:CAAGACTTTGGAGCCGGTTG R:GGAGCAGTTGAGGTCCTTCCC
